# Evaluation of the evolutionary genetics and population structure of *Culex pipiens pallens* in Shandong province, China based on knockdown resistance (*kdr*) mutations and the *mtDNA-COI* gene

**DOI:** 10.1186/s12864-023-09243-2

**Published:** 2023-03-24

**Authors:** Chuanhui Zang, Xuejun Wang, Peng Cheng, Lijuan Liu, Xiuxia Guo, Haifang Wang, Ziwei Lou, Jingjing Lei, Wenqian Wang, Yiting Wang, Maoqing Gong, Hongmei Liu

**Affiliations:** 1 Department of Medical Entomology, Shandong Institute of Parasitic Diseases, Shandong First Medical University & Shandong Academy of Medical Sciences, Jining, 272033 Shandong People’s Republic of China; 2 Shandong Center for Disease Control and Prevention, Jinan, Shandong People’s Republic of China

**Keywords:** *Culex pipiens pallens*, Genetic diversity, Population structure, Shandong province, *Kdr*, *COI*

## Abstract

**Background:**

Mosquitoes are important vectors for a range of diseases, contributing to high rates of morbidity and mortality in the human population. *Culex pipiens pallens* is dominant species of *Culex* mosquito in northern China and a major vector for both West Nile virus and Bancroftian filariasis. Insecticide application were largely applied to control the mosquito-mediated spread of these diseases, contributing to increasing rates of resistance in the mosquito population. The voltage-gated sodium channel (*Vgsc*) gene is the target site of pyrethroids, and mutations in this gene cause knockdown resistance (*kdr*). While these *kdr* mutations are known to be critical to pyrethroid resistance, their evolutionary origins remain poorly understood. Clarifying the origins of these mutations is potential to guide further vector control and disease prevention efforts. Accordingly, the present study was designed to study the evolutionary genetics of *kdr* mutations and their association with the population structure of *Cx. p. pallens* in Shandong province, China.

**Methods:**

Adult *Culex* females were collected from Shandong province and subjected to morphological identification under a dissection microscope. Genomic DNA were extracted from the collected mosquitoes, the *Vgsc* gene were amplified via PCR and sequenced to assess *kdr* allele frequencies, intron polymorphisms, and *kdr* codon evolution. In addition, population genetic diversity and related population characteristics were assessed by amplifying and sequencing the mitochondrial cytochrome C oxidase I (*COI*) gene.

**Results:**

Totally, 263 *Cx. p. pallens* specimens were used for DNA barcoding and sequencing analyses to assess *kdr* allele frequencies in nine *Culex* populations. The *kdr* codon L1014 in the *Vgsc* gene identified two non-synonymous mutations (L1014F and L1014S) in the analyzed population. These mutations were present in the eastern hilly area and west plain region of Shandong Province. However, only L1014F mutation was detected in the southern mountainous area and Dongying city of Shandong Province, where the mutation frequency was low. Compared to other cities, population in Qingdao revealed significant genetic differentiation. Spatial *kdr* mutation patterns are likely attributable to some combination of prolonged insecticide-mediated selection coupled with the genetic isolation of these mosquito populations.

**Conclusions:**

These data suggest that multiple *kdr* alleles associated with insecticide resistance are present within the *Cx. p. pallens* populations of Shandong Province, China. The geographical distributions of *kdr* mutations in this province are likely that the result of prolonged and extensive insecticide application in agricultural contexts together with frequent mosquito population migrations. In contrast, the low-frequency *kdr* mutation detected in central Shandong Province populations may originate from the limited selection pressure in this area and the relative genetic isolation. Overall, the study compares the genetic patterns revealed by a functional gene with a neutral marker and demonstrates the combined impact of demographic and selection factors on population structure.

## Background

Mosquitoes are important vectors for a wide range of pathogenic organisms [1]. *Culex* species are the most common mosquitos in the world, contributing to pathogen emergence and spread through their ability to consume blood meals from humans and animals [2]. Rates of *Culex-*mediated disease outbreaks are becoming increasingly frequent, as in the case of the 2019 outbreak of Eastern equine encephalitis virus (EEEV) in the United States, which caused 34 infections and 11 deaths [3]. *Culex* mosquitos facilitate these spillover events in which EEEV and other deadly diseases are transmitted into humans, horses, and other so-called ‘dead end’ host species [4]. Predicting these outbreaks, however, remains very challenging owing to differences associated with monitoring and predicting mosquito distributions, environmental conditions, human activity, and reservoir-host interactions [5]. In northern China, the most prevalent domestic species of mosquito is *Cx. p. pallens*, which serves as the primary vector for bancroftian filariasis in Shandong Province [6], in addition to potentially facilitating West Nile Virus (WNV) transmission in some areas of China [7]. Studies of *Cx. p. pallens* population genetic structure and evolutionary genetics is potential to inform efforts to prevent vector-borne disease spread and to better evaluate the disease risk in different areas.

Insecticide application is the predominant mode of disease control at present [8, 9]. However, excessive and widespread use has provoked the emergence of high levels of insecticide resistance which are defined by the Insecticide Resistance Action Committee (IRAC) as “the selection of a heritable characteristic in an insect population resulting in the repeated failure of an insecticide product to provide the intended level of control when used as recommended ”[10, 11]. Such resistance continues to hamper efforts to limit the risk of mosquito-borne disease owing to their high levels of rapid toxicity in target insect populations together with relatively limited mammalian toxicity, pyrethroid insecticides have been frequently employed for both indoor and outdoor spraying aimed at controlling mosquito populations in China since the 1980s [12, 13]. *Vgsc* mutation-mediated knockdown resistance is among the leading causes of pyrethroid resistance [14]. Liu et al. explored the relationship between insecticide resistance and *kdr* mutations among mosquito populations in Shandong province, revealing a positive correlation between *kdr* mutations and mosquito survival rate, suggesting that analyses of *kdr* mutations can offer value as a biomarker for studies of pyrethroid resistance in *Cx. p. pallens* populations [15].

It is worth nFoting that some seemingly contradictory results have been reported in prior studies. For one, there is some level of disagreement between studies screening mosquitoes for resistance and studying the role of knockdown resistance in the development of resistance. For example, Su et al. applied deltamethrin to a sensitive population of *Aedes albopictus* to screen for resistance and found that the F1534S mutation was significantly associated with early resistance in this species, and that when resistance rates were high, a novel F1534L mutation was closely associated with resistance [16]. When screening a natural *Cx. p. pallens* population using pyrethroids, Shi et al. found *kdr* to be a major contributor to the early stages of resistance development, with a positive correlation between L1014F mutation frequency and resistance levels together with reductions in *kdr* gene polymorphisms such that the final population only contained a single haplotype [17]. At present, evolutionists believe that the above results are not truly contradictory, as the former is the result of de novo mutations at the target site under the selective pressure of insecticides, while the latter is the result of standing variation screening. Second, diametrically opposing results can also occur when monitoring wild populations. For example, Gao et al. suggested that the I1532T mutation was negatively correlated only with deltamethrin resistance in *Ae. albopictus* [18], while Liu et al. provided the first demonstration that the I1532T mutation was positively correlated with the deltamethrin-resistant phenotype [19]. This may be caused by the different resistance levels observed in wild populations. Researchers believe that mutation frequencies associated with drug resistance phenotypes caused by single-gene mutations increase exponentially. During the initial stage, the incidence of these mutations remains very low such that they are almost undetectable. However, when the resistance reaches a certain level, this mutation frequency will increase rapidly. In addition, knockdown resistance genes can also be affected by multi-site mutations. For example, the V1016G mutation can induce resistance alone, while the presence of both V1016G and S989P can induce stronger resistance. The V1016I does not directly cause resistance but can enhance resistance phenotypes associated with the F1534C mutation. The insecticide resistance phenotypes of mosquitoes are the result of multiple genes or multiple loci mutations in the same gene, together with joint action and long-term inheritance. Therefore, *kdr* mutation polymorphisms, frequencies, and haplotypes are used to trace the origins and development of resistance [20]. Knowledge of mosquito pyrethroid resistance characteristics would offer important guidance for vector control efforts. Detailed genetic analyses of the loci associated with insecticide resistance in particular local populations would also provide insight into the different mechanisms that contribute to such insecticide resistance in that region [21]. While *kdr* mutations are known to be key drivers of resistance to pyrethroid, the precise evolutionary origins of these mutations and their development in *Cx. p. pallens* populations from Shandong province remain to be characterized.

The present study was conducted to assess the patterns of *kdr* allele distribution among *Cx. p. pallens* in Shandong province, to determine whether these mutations were the result of one or more evolutionary origins, and to evaluate the impact of geographical isolation and demographic history on *kdr* mutation evolution in this mosquito population. Analyses of whether resistance-related mutations in a population are derived from a single emergence event and subsequent spreading or evolved independently on multiple occasions can highlight the relative importance that migration and mutation rates play in the dynamics of insecticide resistance. By analyzing neutral biomarker genes and assessing the genetic variation therein, it is possible to gain additional insight into demographic-related population structure. Mitochondrial DNA (mtDNA) markers have emerged as popular targets for analysis when studying genetic diversity and population structure given that they are maternally inherited, do not undergo recombination, are highly variable, and thus necessitate a relatively small target population to facilitate robust analyses [22, 23]. The present study was developed to assess neighboring *kdr* introns in the *Vgsc* gene while utilizing *COI* gene as a marker in order to simultaneously study the evolutionary history of *kdr* mutations and the population structure of *Cx. p. pallens* communities in Shandong Province, China.

## Methods

### Sample collection

Shandong Province is located in Eastern China along the lower reaches of the Yellow River, serving as a primary coastal province. The region exhibits a temperate monsoon climate that is often warm and rainy, making it well-suited to mosquito breeding. *Cx. p. pallens* breeding primarily takes place in moderately polluted standing bodies of water located near to human settlements. For this study, adult female *Cx. p. pallens* specimens were collected from Zibo and Linyi in the southern mountainous area of Shandong; Rizhao, Yantai, and Qingdao in the eastern hilly area of Shandong; and Heze, Dongying, Dezhou, and Liaocheng in the northwest plain region of Shandong from 2021 to 2022 (Table 1). Collected specimens were stored in 100% ethanol at − 80 °C. DNA was extracted from individual mosquitos with the Cador® Pathogen 96 QIAcube® HT Kit based on provided directions and stored at − 80 °C.

**Table 1:** Summary of *Cx. p. pallens* specimen collection sites in Shandong Province

Site ID	Latitude (N)	Longitude (E)	Collection date	Life-stages analysed	Spedmen genotyped
DZ	37°45′	116°31′	8-Jun	adult	57
LC	36°46′	115°98′	4-Jul	adult	30
HZ	35°25′	115°47′	12-Aug	adult	67
DY	37°46′	118°49′	22-Aug	adult	15
ZB	36°81′	118°04′	12-Jun	adult	7
LY	35°07′	118°33′	19-Jul	adult	11
YT	37°54′	121°39′	11-Jul	adult	33
QD	36°09′	120°37′	6-Aug	adult	25
RZ	35°42′	119°46′	22-Jul	adult	15

*DZ* Dezhou, *LC* Liaocheng, *HZ* Heze, *DY* Dongying, *ZB* Zibo; *LY* Linyi, *YT* Yantai, *QD* Qingdao, *RZ* Rizhao

### COI gene sequencing

The 651 bp *COI* gene was amplified with the LCOI490 (5′-GGT CAA CAA ATC ATA AAG ATA TTG G-3′) and HCO2198 (5′-TAA ACT TCA GGG TGA CCA AAA AAT CA-3′) primers via PCR. Each PCR reaction contained 12.5 μl of GoTaq Green Master Mix, 1 μl of each primer (10 μmol/l), 2 μl of template DNA, and nuclease-free water to a final volume of 25 μl. Thermocycler settings were: 94 °C for 1 min; 5 cycles of 94 °C for 40 s, 45 °C for 40 s, and 72 °C for 1 min; 30 cycles of 94 °C for 40 s, 53 °C for 40 s, and 72 °C for 1 min; and 72 °C for 5 min. Amplicons were electrophoretically separated and directly sequenced with the Big-Dye kit (Sangon Biotech Co., Ltd., Shanghai, China).

### Vgsc gene sequencing

The amplification of 521 bp of the *Vgsc* gene including the *kdr* codon at position 1014 was conducted with the *kdr*-F (5′-C CT GCC ACG GTG GAA CTT C-3′)and *kdr*-R (5′-GGA CAA AAG CAA GGC TAA GAA-3′) and primers each PCR reaction contained 12.5 μl of GoTaq Green Master Mix, 1 μl of each primer (10 μmol/l), 2 μl of template DNA, and nuclease-free water to a final volume of 25 μl. Thermocycler settings were: 94 °C for 1 min; 30 cycles of 98 °C for 10 s (denaturation), 55 °C for 15 s, and 68 °C for 30 s; and 68 °C for 1 min. Amplicons were electrophoretically separated and directly sequenced with the Big-Dye kit (Sangon Biotech Co., Ltd., Shanghai, China).

### Data analysis

Compared to *Cx. p. pallens* gene sequences in the GenBank repository using NCBI’s (National Center for Biotechnology Information) online BLAST (Basic Local Alignment Search Tool) algorithmic program. The *COI* gene exhibited a > 99% match to the expected *Cx. p. pallens* gene sequence, and Bioedit v 7.0 was used to align *COI* and *kdr* sequences. DnaSPv6 [24] was used to assess numbers of haplotypes and variable sites, haplotype diversity, and nucleotide diversity. Arlequin 3.5 was used to assess the coefficient of fixation (*Fst*) and gene flow (*Nm*) among groups. Departures from the expected neutral DNA sequence variability were detected through statistical neutrality testing [25]. Tajima’s *D* [26] is based on differences between estimated numbers of segregating sites and average numbers of pairwise differences. Intraspecific molecular polymorphism data are necessary to conduct the *D* and *F* tests developed by Fu & Li, with the latter being based on haplotype frequency distributions [27]. All calculations were performed using DnaSPv6 [24], as were mismatch distribution analyses. PopART was used to construct a network evolution tree with the Neighbor-Joining Network model passed on *kdr* haplotypes. MEGA v.7.0 was used to construct a Neighbor-Joining phylogenetic tree model based upon genetic distance values [28].

## Results

PCR amplification of the *Vgsc* gene in these *Cx. p. pallens* specimens generated a 521 bp PCR fragment. The sequence data for this gene were analyzed to assess *kdr* allele frequency distributions, *kdr* codon evolution, polymorphisms in the neighboring intron downstream of the *kdr* codon, and population genetic diversity. PCR amplification of the *COI* gene generated a 651 bp PCR fragment. As the mitochondrial genome is maternally inherited and haploid, drift towards different haplotype frequencies will occur more rapidly within isolated populations, resulting in differentiation roughly twice that observed for nuclear markers. Accordingly, these mtDNA sequences were analyzed to assess population variations, population expansion, spatial population structure, genetic differentiation, and patterns of gene flow.

### Kdr allele frequency distributions

**Fig. 1:**
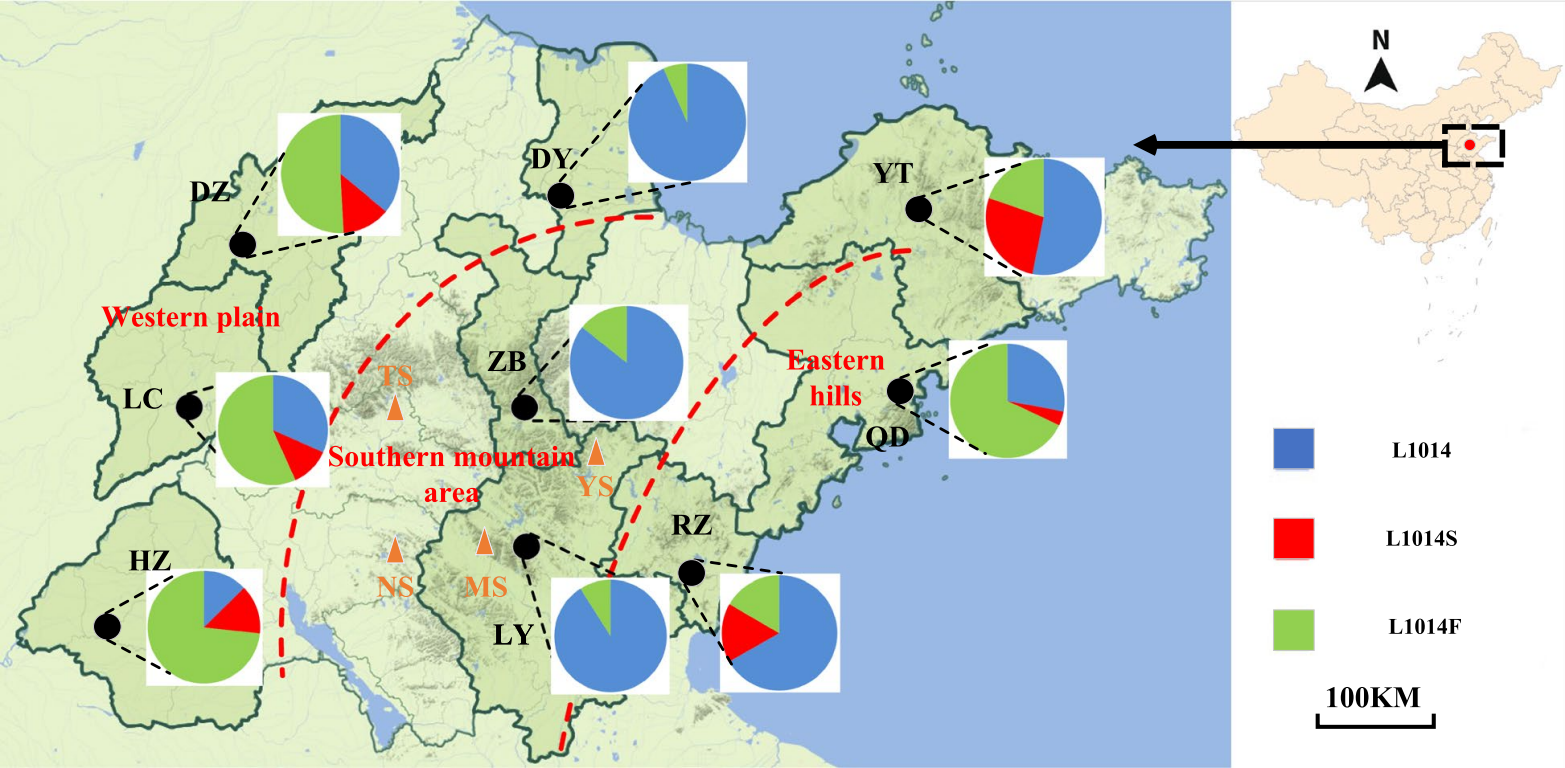
Distribution and frequency of *kdr* mutations in *Cx. p. pallens* in Shandong province. The blue color in the pie charts represents the wild type *kdr* L1014 haplotype, the green color represents the mutant *kdr* L1014F haplotype and the red color represents the mutant *kdr* L1014S haplotype. DZ(Dezhou); LC(Liaocheng); HZ(Heze); DY(Dongying); ZB (Zibo); LY(Linyi); YT(Yantai); QD(Qingdao); RZ(Rizhao).TS(Taishan); YS(Yishan); MS(Mengshan); NS(Nishan)

The wild-type allele (TTA/L) and the mutant alleles (TTT/F, TTC/F, TCA/S) were detected at codon 1014. A total of eight genotypes, including the wild-type genotype TTA/TTA (L/L), the wild-type/mutant heterozygotes TTA/TTT (L/F), TTA/TCA (L/S), and the mutant genotypes TCA/TCA (S/S), TTT/TTT (F/F), TTC/TTC (F/F), TTC/TTT (F/F) and TTT/TCA (F/S), were detected at codon 1014 (Table 2). The *kdr* mutant allele frequencies were found to vary with geographic location. L1014F and L1014S were observed in the northwest plain region (Dezhou, Liaocheng, Heze) and eastern hilly area (Qingdao, Rizhao, Yantai) of Shandong Province. In the northwest plain region, L1014F was the predominant mutation and L1014S was also common. However, only the L1014F was detected in the southern mountainous area and Dongying city at a low mutational frequency (Fig. 1).

**Table 2:** The *kdr* genotype and allele frequency of phenotypes at codon 1014 determined in *Cx. p. pallens* populations from Shandong Province

Site	*n*	L1014 genotype								Allele frequencies (%)		
		TCA/TCA	TTA/TTA	TTT/TTT	TTC/TTC	TTA/TTT	TTA/TCA	TTT/TCA	TTC/TTT	L1014	L1014F	L1014S
DZ	57	3	12	20	1	12	5	4	0	0.359	0.508	0.131
LC	30	2	5	11	1	8	1	2	0	0.316	0.566	0.116
HZ	67	4	4	41	0	7	2	9	0	0.126	0.731	0.141
DY	15	0	13	0	0	2	0	0	0	0.933	0.066	0
ZB	7	0	6	1	0	0	0	0	0	0.857	0.142	0
LY	11	0	9	0	0	2	0	0	0	0.909	0.090	0
YT	33	8	14	1	0	6	1	1	2	0.530	0.196	0.272
QD	25	0	6	15	0	2	0	2	0	0.280	0.680	0.040
RZ	15	2	8	1	0	3	1	0	0	0.666	0.166	0.166

*DZ* Dezhou, *LC* Liaocheng, *HZ* Heze, *DY* Dongying, *ZB* Zibo, *LY* Linyi, *YT* Yantai, *QD* Qingdao, *RZ* Rizhao

L: TTA; F: TTT/TTC; S: TCA

### Kdr haplotype diversity

In total, 28 polymorphic sites were identified in the study. Genetic diversity indices and neutrality tests (Fu’s *Fs* and Tajima’s *D*) were performed using the neighboring intron downstream of the *kdr* allele in *Cx. p. pallens,* with 520 *kdr* intron sequences being included in these analyses. As these polymorphic sites exhibited heterozygous phenotypes, a phase analysis approach was used for haplotype predictions, leading to the detection of 31 haplotypes from 9 populations (Table 3). Overall respective haplotype diversity (*Hd*) and nucleotide diversity (*Pi*) values were 0.664 and 0.0521. The Heze populations exhibited low *Hd* (0.351) and *Pi* (0.01261), whereas these values were high in other populations. No significant departures from neutrality were detected in any of these populations using Tajima’s *D* test, whereas Fu’s *F* test detected significant departures in the Qingdao and Zibo populations, with positive *F* test results indicating low levels of high-frequency populations consistent with a population size reduction and/or balancing selection (Table 3).

**Table 3:** Polymorphism of *kdr* intron and neutrality test of 9 *Cx. p. pallens* populations

Site	*n*	*s*	*Pi* (×10^−2^)	*h*	*Hd*	Tajima’s *D* Test	Fu’s *Fs* Test	*P*	Fu & Li’s D* Test	Fu & Li’s F* Test
DZ	114	21	6.165	12	0.725	−0.449	1.743	0.091	1.1621	0.6372
LC	60	17	4.196	10	0.656	−0.136	0.637	0.152	−0.129	− 0.156
HZ	134	11	1.261	5	0.351	−0.746	1.931	0.135	−2.067	−1.898
DY	30	21	10.315	15	0.894	−0.049	− 1.535	0.085	− 0.097	− 0.096
ZB	14	27	7.048	4	0.747	−0.158	6.639	0.007 *	1.5959**	1.2831
LY	22	15	7.502	9	0.853	0.0277	0.409	0.19	1.5811**	1.3008
YT	66	19	4.675	15	0.708	−0.371	−1.706	0.067	0.9595	0.5716
QD	50	14	5.137	6	0.559	0.4212	4.477	0.023*	0.7212	0.7329
RZ	30	16	5.816	11	0.834	0.2153	−0.394	0.152	1.2585	1.0899

*DZ* Dezhou, *LC* Liaocheng, *HZ* Heze, *DY* Dongying, *ZB* Zibo, *LY* Linyi, *YT* Yantai, *QD* Qingdao, *RZ* Rizhao. *n* = number of genes (two per individuals), *s* = number of polymorphic (i.e., segregating) sites, *Pi* = nucleotide diversity, *h* = number of haplotypes, *Hd* = haplotype diversity. *P < 0.05; **P < 0.01

### Genealogical analyses of kdr mutations

**Fig. 2:**
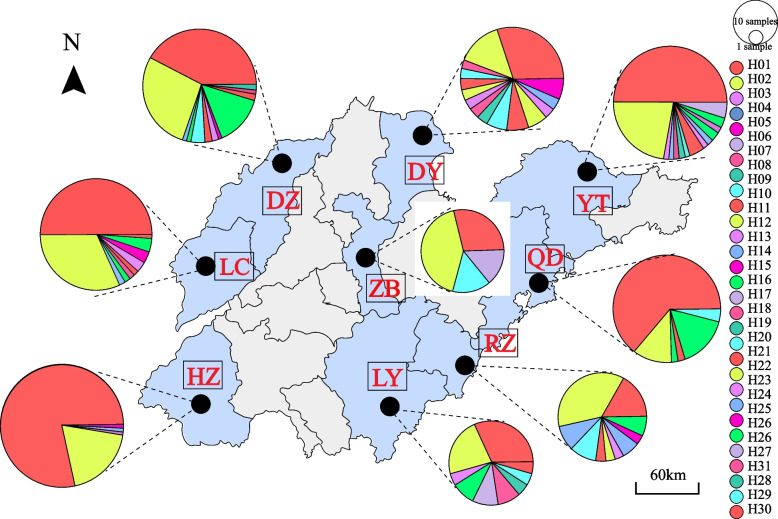
Geographic distribution of Intron haplotypes of *kdr* allele in the 9 *Cx. p. pallens* populations. The area of the circle represents the haplotype frequency, different colors indicate different haplotypes. DZ(Dezhou); LC(Liaocheng); HZ(Heze); DY(Dongying); ZB (Zibo); LY(Linyi); YT(Yantai); QD(Qingdao); RZ(Rizhao)

To estimate the evolutionary relationship of *kdr* mutations, 31 haplotypes were identified in this study. Two haplotypes were predominant in the populations: H01 (52.5%) and H02 (23.8%) (Fig. 2). The other detected haplotypes exhibited only limited geographic distributions or were unique to individual populations. Overall, there were 13 unique haplotypes and 18 shared haplotypes, with the former accounting for 58.1% of all haplotypes. The H01 and H02 haplotypes were shared among all study sites, while substantial haplotype sharing was also observed between the Liaocheng and Dezhou populations (including H06, H11, H13, and H15). Many shared haplotypes were also observed among the Qingdao, Dongying, Yantai, and Rizhao populations. In addition to these eastern coastal cities, the Qingdao population exhibited only limited sharing of haplotypes with other regions suggesting that this population is relatively differentiated from other populations, in line with the genetic differentiation coefficient *Fst*. Other than the H01 and H02 haplotypes, only one shared haplotype was present in the Zibo population. The geographically non-adjacent Yantai and Liaocheng populations shared seven haplotypes, with a negative genetic differentiation coefficient *Fst* is − 0.00859, suggesting that no appreciable genetic differentiation and high levels of communication between these populations. Given the limited sampling at each study site and associated time constraints, further analyses will be needed to confirm and expand upon these findings.

**Fig. 3:**
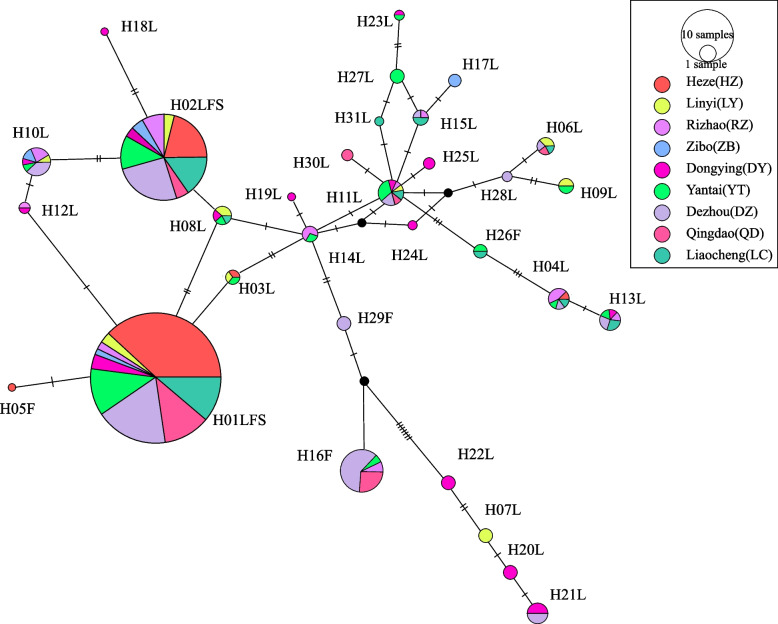
*kdr* haplotype networks showing the genealogical relationship for *Cx. p. pallens*. Each haplotype is represented by a circle with size proportional to its frequency in the sample. Different colors indicate different populations. Lines represent variable asynchronous numbers; black solid circles represent missing haplotypes. The note above the circle referred to the mutation position and base. H: haplotype. H01LFS represent H01-L, H01-F and H01-S; H02LFS represent H02-L, H02-F and H02-S

Network analysis results revealed a complex reticulate pattern and multiple independent mutational events resulting in the observed *kdr* haplotypes (Fig. 3). The H11 haplotype is largely in the center of this network as it is most closely related to the other haplotypes such that it may be a more evolutionarily ancient haplotype that can undergo mutation through one or more steps to yield the other haplotypes. Haplotypes H01-F and H01-S comprise the majority of samples in all populations and may be derived from ancestral H01-L, H08-L, H03-L, and H12-L haplotypes through one or two mutational steps. Likewise, the H02-F and H02-S haplotypes may be derived from the ancestral H02-L, H08-L, H010-L, and H18-L haplotypes through one or two mutational steps. The H05-F haplotype found in Heze stems from H01-L through one mutational step, while the H26-F haplotype from Yantai and Liaocheng is likely to have arisen from H11-L through three mutational steps. The H16-F and H29-F haplotypes are derived from a shared ancestor lacking the *kdr* haplotype. Overall, these genealogical analyses suggest that as few as one mutational step may differentiate between resistant and susceptible phenotypes among *Cx. p. pallens.*

### Analyses of mtDNA sequence variation

**Fig. 4:**
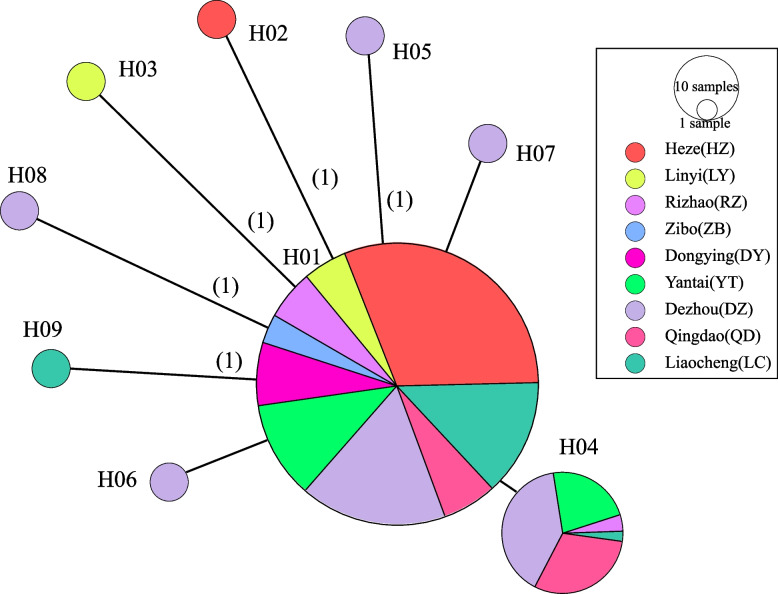
Phylogenetic network of 9 mitochondrial haplotypes of the *COI* gene in *Cx. p. pallens* in Shandong province. The haplotype network graph constructed based on Median-joining method. The size of each circle is proportional to its corresponding frequencies. Different colors indicate different populations. The number above the line referred to variable asynchronous numbers

**Fig. 5:**
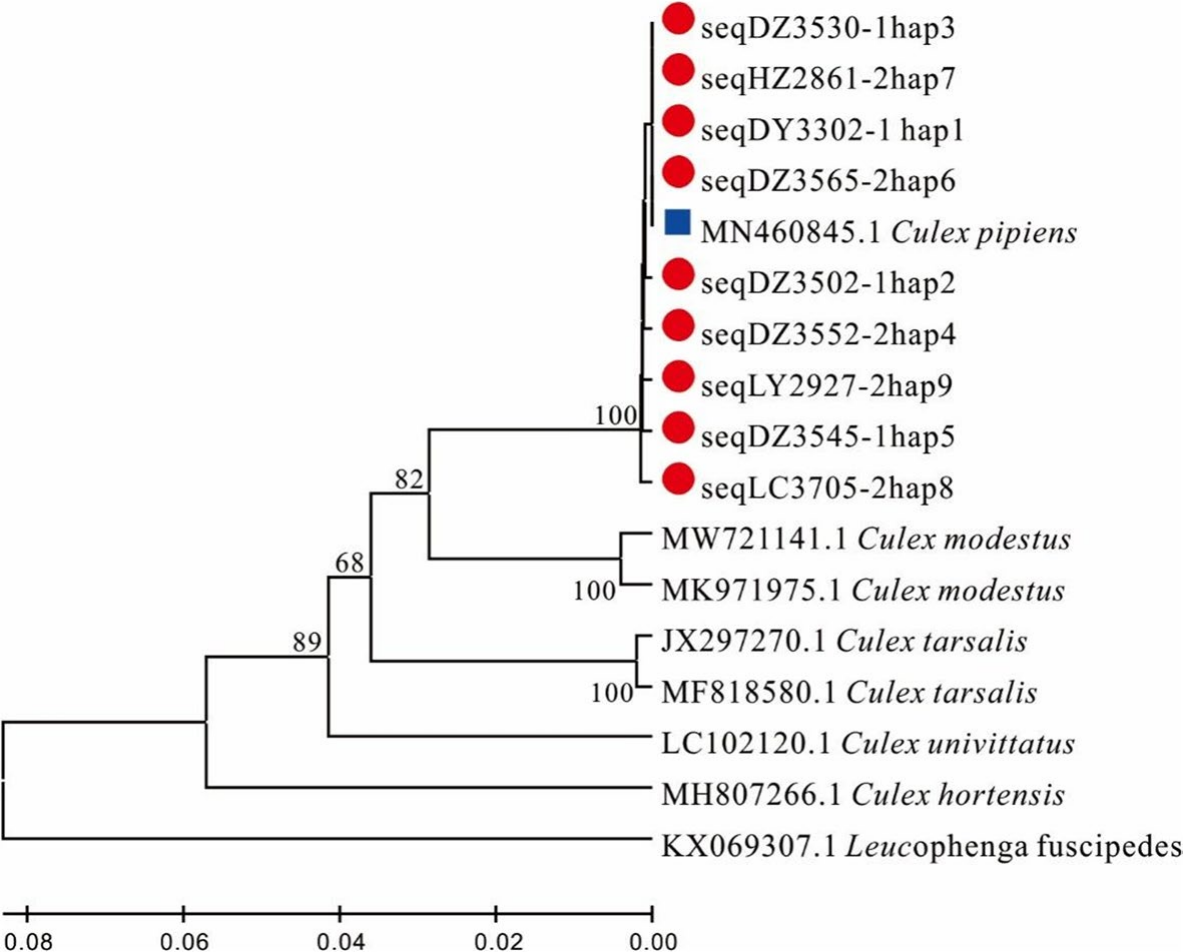
Phylogenetic analysis based on *COI* haplotype variation. Marked with a solid red circle are haplotypes in the current study; others were retrieved from GenBank. Haplotypes marked with a solid blue square are associated with *Cx. p. pallens*. Neighbor-joining trees were constructed via the maximum composite likelihood substitution model using MEGA (version 7.0). Numbers at branches represent bootstrap values of 1000 replicates (values > 50 are shown). The scale-bar shows the number of nucleotide substitutions per site

A total of 520 mtDNA- *COI* sequences were next conducted polymorphism analyses, leading to the identification of 8 variable sites and 9 haplotypes (Table 4). The overall haplotype diversity (*Hd*) and nucleotide diversity (*Pi*) values were 0.306 and 0.00052, respectively. The low haplotype diversity and low nucleotide diversity indicated that different geographic populations may have experienced a “bottleneck effect”. Dezhou population exhibited the highest levels of diversity, whereas the Dongying and Zibo populations were the least diverse. The haplotype network graph constructed based on Median-joining method shows a radiative evolutionary center with a star-shaped distribution (Fig. 4). In total, 2 and 7 shared and unique haplotypes were identified, respectively. Of these, the H01 haplotype was the most widely distributed primitive haplotype, radiating out to one shared haplotype and multiple unique haplotypes. This star-shaped network phylogeny of this network also supports the notion that these *Cx. p. pallens* populations expanded following population bottleneck events, but that these populations are still undergoing expansion. The H04 haplotype was shared among the Rizhao, Qingdao, Dezhou, and Yantai populations. Other haplotypes were only present at low frequencies. H02, H03, and H09 were respectively unique to the Heze, Linyi, and Liaocheng populations, while H05-H08 were unique to the Dezhou population. The observation that these haplotypes were unique to these respective regions may be a consequence of limited sample selection or may be a result of genetic variation among these geographic environments. To better assess the *Cx. p. pallens* population structure and phylogenetic relationships among these populations, a Neighbor-joining (NJ) tree incorporating these 9 *COI* sequence-based haplotypes was constructed (Fig. 5), revealing that all haplotypes clustered in a single branch.

**Table 4:** Polymorphism of *COI* and neutrality test of 9 *Cx. p. pallens* populations

site	*n*	*s*	*Pi* (×10^−4^)	*h*	*Hd*	Tajima’s *D* Test	Fu’s *Fs* Test	*P*	Fu & Li’s D* Test	Fu & Li’s F* Test
DZ	114	5	9.3	6	0.541	−0.837	−2.061	0.072	1.025	0.485
LC	60	2	2.2	3	0.129	−1.186	−2.05	0.095	0.729	0.181
HZ	134	1	0.5	2	0.030	−0.908	−1.742	0.138	0.475	0.062
DY	30	0	0	0	0	/	/	/	0	0
ZB	14	0	0	0	0	/	/	/	0	0
LY	22	1	1.5	2	0.091	−1.162	−0.957	0.239	−1.574	−1.678
YT	66	1	6.7	2	0.403	1.150	1.626	0.315	0.520	0.818
QD	50	1	8.4	2	0.509	1.721	1.981	0.276	0.543	1.020
RZ	30	1	4	2	0.239	0.0626	0.388	0.383	0.594	0.469

*n* = number of genes (two per individuals), *s* = number of polymorphic (i.e., segregating) sites, *Pi* = nucleotide diversity, *h* = number of haplotypes, *Hd* = haplotype diversity. *DZ* Dezhou, *LC* Liaocheng, *HZ* Heze, *DY* Dongying, *ZB* Zibo, *LY* Linyi, *YT* Yantai, *QD* Qingdao, *RZ* Rizhao

### Genetic differentiation among populations

An analysis of molecular variance (AMOVA) revealed that genetic differences of *COI* gene among and within populations respectively accounted for 18.3 and 81.7%, with a fixation index *Fst* value is 0.18293. As such, within-populations variance is the primary source of genetic variation among the *Cx. p. pallens* populations of Shandong Province. The pairwise *Fst* and *Nm* value estimates further highlight genetic differentiation among these populations (Table 5). A range of factors can contribute to the genetic differentiation of populations, including transport or other human activities, landscape barriers, and geographical distances among these populations. *Fst* values for the genetic differentiation index among these different mosquito populations ranged from − 0.00802 to 0.46939. The only negative *Fst* values were those between the Dezhou and Yantai populations. No significant differentiation was observed among 14 pairs of populations (*Fst* < 0.05; 38.9%), while moderate differentiation (0.05 < *Fst* < 0.15), high differentiation (0.15 < *Fst* < 0.25), and very high differentiation (*Fst* > 0.25) were respectively observed among 7 (19.4%), 8 (22.2%), and 7 (19.4%) pairs of populations. The highest *Fst* value was observed between the Qingdao-Heze, Qingdao-Zibo, Qingdao-Dongying populations (Table 5). *Nm* values corresponding to the gene flow among these populations were between − 31.43 and 134.26. Together, these findings were thus consistent with roughly half of the populations exhibiting high or very high levels of genetic differentiation and low levels of gene flow among these populations. Fixed coefficient *Fst* values between pairs of populations ranged between − 0.00802 and 0.46939 (Table 5), and the highest levels of genetic differentiation were observed when comparing the mosquito populations from Qingdao and either Dongying/Zibo (*Fst* = 0.46939; *Nm* = 0.28), owing to the presence of the H4 haplotype in Qingdao and its absence in Dongying/Zibo.

**Table 5:** Mitochondrial DNA based population differentiation for population pairs (estimates of *Fst* below diagonal and *Nm* above diagonal)

	DZ	LC	HZ	DY	ZB	LY	YT	QD	RZ
DZ	–	1.44	0.95	0.9	0.9	1.05	−31.43	3.97	5.99
LC	0.14807	–	16.17	14.5	14.5	24.56	1.23	0.41	7.67
HZ	0.20837	0.01522	–	33	33	134.26	0.76	0.3	2.42
DY	0.21671	0.01695	0.00752	–	1	0	0.71	0.28	2.17
ZB	0.21671	0.01695	0.00752	0	–	0	0.71	0.28	2.17
LY	0.19239	0.01008	0.00186	0	0	–	0.87	0.33	2.99
YT	−0.00802	0.16897	0.24836	0.26154	0.26154	0.22418	–	3.27	6.47
QD	0.05923	0.3779	0.45546	0.46939	0.46939	0.42878	0.07092	–	0.84
RZ	0.04036	0.03157	0.09379	0.10345	0.10345	0.07715	0.03718	0.22891	–

*DZ* Dezhou, *LC* Liaocheng, *HZ* Heze, *DY* Dongying, *ZB* Zibo, *LY* Linyi, *YT* Yantai, *QD* Qingdao, *RZ* Rizhao

### Population demography history

In an effort to infer the demographic history of *Cx. p. pallens* populations in Shandong Province, Tajima’s *D* and Fu’s *Fs* tests were next conducted, with the results and simulated *P*-values being shown in Table 4. Negative Tajima’s *D* and Fu’s *Fs* values were obtained for the Liaocheng, Dezhou, Linyi and Heze populations, suggesting high numbers of low-frequency mutations in these populations and that they are actively undergoing expansion. The frequency distributions for pairwise differences between sequences were also assessed to evaluate historical demographic expansions. Mismatch distributions can be used to visually assess historical population dynamics. Two models correspond to expected DNAsp values, with one assuming that the population size remains constant and the other assuming that the population grows or declines. When observations conform to one of these models, it indicates that the population is in line with the expected model-derived values and the population is constant or shrinking, whereas if the values deviate from expected values this indicates the population is expanding with a single-peak curve. The present results indicated that actual observations were consistent with hypothetical model values, thus indicating that the *Cx. p. pallens* populations in Shandong were in a state of genetic equilibrium.

## Discussion

In the study, patterns of *kdr* mutations associated with insecticide resistance in *Cx. p. pallens* populations were studied by the *Vgsc* gene from mosquito populations in Shandong Province. The origins of these *kdr* mutations were explored through haplotype networks and comparisons of regional variability in mutation frequencies among these populations. The *COI* gene was further used to analyze the population structure in these study sites to clarify the differences among regions, supporting a model in which both geographical isolation and varying levels of selection pressure have shaped these communities.

### Geographical isolation

Based on mtDNA sequencing, significant genetic differentiation was detected between populations from eastern hilly area of Shandong and southern mountainous area of Shandong. When comparing these two regions the *Fst* value was 0.28 consistent with substantial genetic differentiation. Notably, the *Fst* values when comparing the Qingdao population and other populations ranged from 0.059–0.469. *Nm* values were consistent with these findings, reaffirming the higher level of genetic differentiation for the Qingdao population as compared to the other studied populations. This may be a result of the fact that the sampling site was located in the Huangdao district of Qingdao. Historically, Huangdao was an isolated island surrounded by the sea, making travel to inland areas challenging. However, the Qingdao Jiao Zhou Bay Bridge and the Jiao Zhou Bay Subsea Tunnel were completed in 2010 and 2011 respectively. The development of transportation methods has facilitated transportation between Huangdao and other cities, and economic advances continue to contribute to rising transregional, transnational, and transcontinental vessel exchanges. Beginning in 2010, Liu et al. began investigating the association between kdr mutations and insecticide resistance in *Cx. p. pallens* of Shandong province, they found lower frequencies of mutations in Qingdao populations as compared to other study sites. The World Health Organization (WHO) standard resistance bioassay was applied to test insecticides resistance in *Cx. p. pallens*, Huangdao district populations exhibited a mortality rate of 83.8% consistent with preliminary resistance, whereas mortality rates in Jining populations were just 37.3% consistent with high levels of resistance [18]. Compared to studies from a decade ago, *kdr* mutations frequency in Qingdao populations significantly increased in this study, that may be because the Qingdao Jiao Zhou Bay Bridge and the Jiao Zhou Bay Subsea Tunnel were completed, promoting communication among different populations, but the main reason is related to the using of pesticides. The Huangdao port thus provides a potential site not only for the interactions among people and the dissemination of goods and services, but also for the spread of infectious diseases. The Huangdao port is currently an important node in over 150 international routes, engaging in trade with over 450 ports in over 130 countries. Over 60 international long-distance sea routes connect this port to far-flung areas including South Africa, the Black Sea, Europe, the Americas, the Mediterranean Sea, and Australia. Invasive mosquito vector species are being passively transported to new areas by humans for decades. However, current levels of mobility with a worldwide increased flow of people and commodities have raised the risk of the introduction of invasive mosquito species considerably. Mosquito surveillance in maritime ports of entry is crucial to allow the early detection of invasive mosquito species [29]. A total of 136 whole blood samples were collected from malaria suspect cases at Qingdao port from 2014 to 2019. Forty-six malaria cases were confirmed with the rate 33.82%. There were no local malaria cases in Qingdao City, the prevention and control of imported malaria is the focus of the future work [30]. Accordingly, there is a strong need to develop appropriate port quarantine protocols, to improve the health services provided in port cities, and to improve public health emergency response measures to ensure public safety.

### The impact of insecticide-associated selection

Heze is situated in southwestern Shandong Province in the lower reaches of the Yellow River, and contains extensive areas of flat terrain with deep soil that is well-suited to agricultural use. It is a primary site of grain production in this province and bordering Anhui, Henan, and Jiangsu provinces to which good transportation is available. Vector-borne disease outbreaks have been historically common in Heze, and it was the last city in Shandong Province to eliminate new malaria cases [31]. Relative to other analyzed populations, the *Cx. p. pallens* population from Heze exhibited lower levels of genetic variation in the intronic regions associated with and a high frequency of *kdr* mutation (L1014F), indicating that the low levels of intronic variation may be attributable to a selective sweep, referring to a rapid increase in the frequency of a given allele as a consequence of directional selection, tied to high levels of intensive insecticide application and/or from the fixation of this resistance genotype. During a selective sweep, linked alleles in regions flanking the allele subject to selective pressure can also rise in frequency, contributing to a drop in overall genetic variation [32]. Comparisons of the DNA sequences flanking the *kdr* L1014 codon indicated a high degree of genetic differentiation between the Heze population and other analyzed populations consistent with strong local selection for this resistance allele in Heze. In contrast, these mutational frequencies were low in Dongying, Linyi, and Zibo, with only one mutant allele (L1014F) being present. Zibo is south of the Tai-Yi Mountain range, Linyi is adjacent to Zibo. From north to south, the three main mountains in Linyi are the Yi, Meng, and Ni mountains, which probably due to interrupted gene flow from eastern hilly or northwest plain region to southern mountainous area, caused by geographical isolations. While Dongying is in the northwest plain regions of Shandong province, it is near the end of the Yellow River and the coast of the Bohai sea where the flow of the Yellow River into the sea slows, forming a large delta that has arisen through the rapid deposition of high levels of silt sediment, resulting in shallow belowground water levels and extremely salinized soil that contributes to high levels of ecological fragility. On the one hand, currently, the saline-alkali land area in Dongying city is 350 km2, most crops cannot grow in saline-alkali soils, agricultural cultivation is relatively poor [33, 34]. Compared to other city, pesticides used less. On the other hand, saline-alkali stress reduces soil bacterial community diversity and soil enzyme activities and affects the metabolism of pesticides [35, 36]. Mosquito gut microbes may participate in the resistance of the host to insecticides to alleviate the effects of insecticides on the host [37]. Saline-alkali soil may affect the development of mosquito resistance, but the involved microorganism needs further research. When analyzing *kdr* codon L1014 sequences, significant departures from neutrality were observed in Linyi and Zibo, resulting from population bottleneck events. Rates of agricultural insecticide application in Dongying may be relatively low, potentially explaining the lack of insecticide resistance alleles in this population. Of the different neutrality tests, Fu’s *Fs* statistic exhibits the greatest power when detecting departures from neutrality based on a genetic hitchhiking model [32]. High Fu’s *Fs* measures for most of the study sites in Shandong province may be attributable to the high levels of insecticide use and strong selection placed on both the *kdr* locus and other regions of the genome. Over the past 50 years, insecticides have been widely used in agricultural settings and for vector control throughout central China [22]. The rapidly rising rates of resistance to these insecticides and the widespread *kdr* mutation distributions observed among these *Cx. p. pallens* populations underscore the need for the establishment of resistance surveillance plans and efforts to better manage insecticide efficacy.

### The multiple origins of kdr mutations

The results of genealogical analyses of the intron downstream of the *kdr* codon together with the observed patterns of geographic distribution strongly indicate that these *kdr* mutations are derived from multiple mutational events rather than a single origin. Two non-synonymous codon L1014 mutations were detected among analyzed mosquito populations. While just one of these mutations was evident in central Shandong Province, both were found in the western and eastern coastal regions of the province. This is consistent with prior *kdr* genotyping results published for *Cx. p. pallens,* which revealed that the L1014F mutation was widely distributed throughout the Shandong region [38]. Two potential models can explain the emergence of *kdr* mutations. Specifically, these mutations may have arisen once and then spread over time, or they may have independently evolved in multiple geographically distinct populations. At least two independent mutational events were identified as having given rise to the L1014F or L1014S haplotypes through a single mutational step (Fig. 3). This is consistent with observations in *Anopheles gambiae*, which is the primary vector for malaria in Africa, in which a minimum of 4–5 independent non-synonymous mutations have been documented at position L1014 of the *Vgsc* gene [39]. Some *Vgsc* channel gene mutational events have been shown to be derived from a single mutation from a common ancestor, whereas others result from two mutational steps [40]. Network analyses revealed that just one mutational step can change H01-F/H01-S to H05-F (Fig. 3). Given these findings, H05-F may correspond to a *kdr* allele that evolved through sequential progression from H01-F/H01-S. Other species of insects have similarly been shown to exhibit multiple point mutation-mediated origins of resistance alleles in the voltage-gated sodium channel gene [39, 41], and multiple *kdr* mutation origins have been reported in *Musca domestica* and in the aphid *Myzus persicae* [42]. Moreover, *kdr* mutations of multiple origins are common among *Culicinae* mosquitoes, with relevant reports in *Anopheles gambiae*, *Anopheles sinensis, and Aedes aegypti* having been published to date [43, 44].

## Conclusions

The geographic distribution of *kdr* haplotypes should reflect the interplay between the evolutionary forces of mutation, gene flow and selection. Overall, these data suggest that the multiple origins of *kdr* insecticide resistance alleles observed in *Cx. p. pallens* from Shandong Province are attributable to the prolonged and intensive application of insecticides in agricultural contexts. In contrast, the populations lacking these *kdr* mutations are more likely to be genetically isolated and free of substantial selection pressure. By comparing genetic patterns for both a gene functionally associated with insecticide resistance and a neutral marker gene, these findings simultaneously offer insight into the combined effects of selection and demographic factors on the resultant *Cx. p. pallens* population structure. In addition, more ecological studies are needed, relating levels of insecticide resistance with the genotypic composition at the *kdr* locus and with the analysis of other resistance mechanisms (e.g., metabolic, behavioral). These analyses would provide both valuable insight into the molecular evolution of pyrethroid resistance and a foundation for subsequent vector control efforts aimed at controlling the spread of mosquito-borne illness.

## Data Availability

All data generated or analyzed during this study are included in this published article and its additional files.

## Abbreviations

*Vgsc*: Voltage-gated sodium channel
*kdr*: Knockdown resistance
WNV: West Nile virus
EEEV: Eastern equine encephalitis virus
IRAC: Insecticide Resistance Action Committee
*Hd*: Haplotype diversity
*Pi*: Nucleotide diversity
*COI*: Mitochondrial cytochrome C oxidase I gene
